# Differentiation stage-specific expression of transcriptional regulators for epithelial mesenchymal transition in dentate granule progenitors

**DOI:** 10.3389/fnins.2024.1425849

**Published:** 2024-08-29

**Authors:** Kyoji Ohyama, Hiroshi M. Shinohara, Natsumi Takayama, Rina Ogawa, Shoichiro Omura, Mio Hayashida, Tokiharu Takahashi

**Affiliations:** Department of Histology and Neuroanatomy, Tokyo Medical University, Tokyo, Japan

**Keywords:** EMT, Zeb1, Scratch2, Nkx6-2, dentate gyrus, granule cell differentiation

## Abstract

During the development of the mouse dentate gyrus (DG), granule neuronal progenitors (GNPs) arise from glial fibrillary acidic protein (GFAP)-expressing neural stem cells in the dentate notch. However, the transcriptional regulators that control their stepwise differentiation remain poorly defined. Since neurogenesis involves epithelial-to-mesenchymal transition (EMT)-like processes, we investigated the spatio-temporal expression profiles of the EMT transcription factors Zeb1, Scratch2 (Scrt2) and Nkx6-2 in relation to known GNP markers. Our results show that Zeb1 and Scrt2 exhibit sequential, but partially overlapping expression across embryonic and postnatal stages of GNP differentiation. Zeb1 is highly enriched in *gfap*-GFP+/Sox2+ neural stem/progenitor pools and subsets of Tbr2+/Prox1+/NeuroD+ intermediate GNPs, whereas Scrt2 predominates in Tbr2+/Prox1+/NeuroD+ GNPs. Strikingly, the neuronal EMT regulator Nkx6-2 shows selective expression in postnatal Tbr2+/Prox1+ GNPs, but it is excluded from embryonic counterparts. This temporally coordinated yet distinct expression of Zeb1, Scrt2 and Nkx6-2 reveals discrete transcriptional programs orchestrating GNP differentiation and neurogenic progression at embryonic versus postnatal stages of DG neurogenesis.

## Introduction

In the developing central nervous system, the expression of transcription factors (TFs) not only defines progenitor cell types but also orchestrates their differentiation. A previous study showed that *gfap*-GFP+ cells arising around the dentate notch (DN) first express Sox2 and give rise to Tbr2+ intermediate progenitors, which then contribute to the formation of the Prox1+ granule neuronal cell layer (Seki et al., 2014). Some *gfap*-GFP+ cells maintain their stemness, at later stages, and contribute to neural stem/progenitor cells in the subgranular zone (SGZ) of the postnatal DG (Matsue et al., 2017). Prox1 not only regulates a granule cell fate over a pyramidal neuronal fate in the hippocampus, but also neuronal differentiation and maintenance of post-mitotic states (Iwano et al., 2012). To better understand the mechanism for the progressive differentiation of dentate granule neurons, it is important to identify in more detail the temporally regulated expression of TFs in the granule neuronal lineage.

Emerging evidence suggests that different epithelial mesenchymal transition (EMT)-TFs play distinct roles in controlling stemness and the onset of neuronal differentiation (Singh and Solecki, 2015). We have recently shown that pSmad3, a key mediator of EMT is expressed in DG stem/progenitors and RGL cells in the developing DG (Ohyama et al., 2023). Zeb1, downstream of pSmad3, contributes to maintaining the stemness of cancer-initiating cells (CICs) by inhibiting cellular senescence (Ansieau et al., 2008; Chaffer et al., 2013). Zeb1 also controls the stemness of GNPs in the cerebellum through the downregulation of cell polarity genes (Singh et al., 2016). Conversely, inhibition of Zeb1 leads to upregulation of cell polarity genes, resulting in cerebellar granule neuronal differentiation (Singh et al., 2016). Another EMT-TF, Snail, regulates the number of neural progenitors throughout life (Zander et al., 2014). Scrt2, a member of the Snail family TFs, inhibits cell cycle re-entry through the regulation of miR25, de-represses the expression of the cyclin-dependent kinase inhibitor p57, thereby triggering the onset of neuronal differentiation in the developing spinal cord (Rodríguez-Aznar et al., 2013). Scrt2 also controls the onset of neuronal migration in the mouse cerebral cortex (Itoh et al., 2013). In addition, Nkx6-2 has been identified as a neuronal EMT-TF (Liang et al., 2016). These studies suggest that different EMT-TFs play time-dependent roles during neuronal differentiation.

Here, we examined the expression patterns of the EMT-TF Zeb1, Scrt2, and Nkx6-2 in the developing DG, and compared them with the temporal expression profiles of previously identified TFs that are expressed in the dentate GNPs.

## Materials and methods

### Animals

*Gfap*-GFP mice on a C57BL6/NCrl background (Suzuki et al., 2003) were housed under standard conditions (12 h light/dark cycle) at the animal care facility of Tokyo Medical University. All experiments were carried out in accordance with the guidelines of the Institutional Animal Care and Use Committees and conformed to the National Institutes of Health Guide for the Care and Use of Laboratory Animals (NIH Publication No. 80-23), revised in 1996. Every effort has been made to minimize the number of animals used and their suffering. Embryos and pups of *gfap*-GFP transgenic and C57BL6 wild-type mice were used. The day on which a vaginal plug was found was referred to as embryonic day 0.5 (E0.5) and the day of birth was referred to as postnatal day 0.5 (P0.5).

### Antibodies

The following primary antibodies were used: anti-GFP (abcam, ab13970, 1:5,000); anti-Lhx1/5 mouse IgG (DSHB, 4F2, 1:50); anti-Neuro D goat IgG (N-19) (Santa Cruz, sc-1084, RRID:AB_630922, 1:1,000); anti-Nkx6-2 (Millipore, ABN1455); anti-p73 goat IgG (Santa Cruz, sc-9651, 1:250); anti-Prox1 goat IgG (R&D, AF2727, 1:1,000); anti-Tbr2/EOMES rat IgG conjugated to Alexa 660 (eBioscience, 50-4375-82, 1:1,000); mouse anti-reelin (Millipore, MAB5364, clone G10, 1:1,000); anti-Scratch2 (Scrt2) rabbit polyclonal antibody (gift of Y Gotoh; 1:1,000); anti-Sox2 goat IgG (R&D, AF2018, 1:1,000); anti-Zeb1 rabbit IgG (Thermo Fischer Scientific/Sigma HPA027524, 1:1,000); anti-Zeb1 mouse IgG (eBioscience, 14-9741-80, 1:250). The following secondary antibodies were used: Alexa Fluor 555 plus donkey anti-rabbit IgG (Thermo Fisher, A32794); Alexa Fluor 488 donkey anti-chick IgY (Thermo Fisher, A78948); Alexa Fluor 488 plus donkey anti-goat IgG (Thermo Fisher, A32814); Alexa Fluor 488 plus donkey anti-mouse IgG (Thermo Fisher, A32766); Alexa Fluor 647 donkey anti-rat IgG (Thermo Fisher, A48272).

### Immunohistochemistry

Cryosections were processed for immunohistochemistry as previously described (Ohyama et al., 2023). Briefly, embryos and pups of *gfap*-GFP transgenic mice and sibling controls were fixed in 4% PFA at 4°C overnight. After fixation, samples were immersed in 30% sucrose in 0.1 M PB at 4°C overnight and then embedded in OCT compound. Cryosections of the hippocampus in the coronal plane were incubated with primary antibodies at 4°C overnight. After three washes with PBS, sections were incubated with secondary antibodies for 45 min at room temperature. After three washes with PBS, the sections were coverslipped with Vectashield (Vector Laboratories, H-1200, CA). Images were captured using a Zeiss LSM700 confocal microscope with Zeiss Image Browser, ZEN software (Zeiss, Thomwood, NY). Images were corrected for brightness and contrast and composited using Adobe Photoshop CS6 (San Jose, CA). Mice (*n* = 3–5) were examined and, for quantification of some experiments, at least 6 sections were analyzed for each using Fiji of image J. Mean ± SE is given in the results.

### Quantification using Fiji

To quantify marker expression, we used Fiji-ImageJ software. First, we opened the TIFF file containing the red (e.g., Zeb1) and green (e.g., Sox2) fluorescence using Fiji. We set the threshold color to white to distinguish the area of interest and adjusted the brightness as needed to improve contrast and reduce the background noise. Using the Hue sliders, we isolated the specific signal, such as Zeb1+Sox2+ cells and used the Analyse>Measure function to count these cells. We repeated this process to count Sox2+ cells. The resulting cell counts were then transferred to an Excel spreadsheet where we calculated the percentage of, for example, Zeb1+Sox2+ cells relative to Zeb1+ cells. This approach was taken by two individuals through blinding and ensured consistent and accurate quantification of marker expression across samples.

## Results

### Temporally ordered expression of Sox2, Tbr2, NeuroD, and Prox1 during the differentiation of *gfap*-GFP+ GNPs into dentate granule neurons

**Figure 1:**
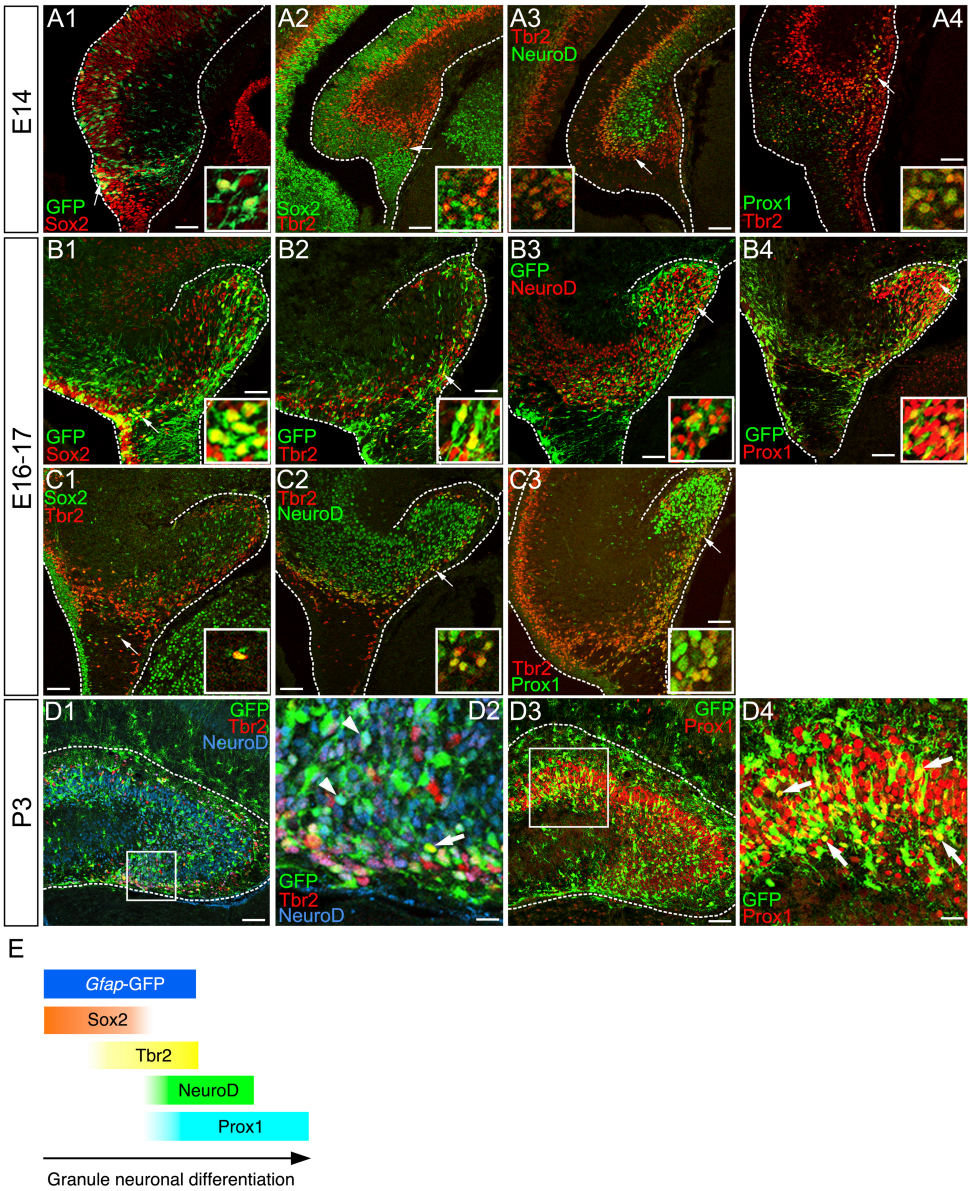
Temporally coordinated expression of Sox2, Tbr2, NueroD, and Prox1 in *gfap*-GFP+ GNPs and differentiating granule neurons *Gfap*-GFP+/Sox2+ progenitor cells were found at the dentate neuroepithelium at E14 (arrow in **A1**). Sox2 expression was found at the VZ. Tbr2+ GNPs are then observed (red, arrow in **A2**). Tbr2 expression is followed by NeuroD expression **(A3)**. Tbr2+/NeuroD+ GNPs are found (arrow, **A3**). The Tbr2+ GNPs (red) migrate toward the DG primordium and co-express Prox1 (green), a marker for early differentiating dentate granule neurons (arrow in **A4**). *Gfap*-GFP+ progenitors sequentially express Sox2, Tbr2, NeuroD1, and then, Prox1 in the developing DG at E16 (arrows in **B1–B4**, respectively). Some Sox2+/Tbr2+, Tbr2+/NeuroD+, and Tbr2+/Prox1+ GNPs are found. Similarly, *gfap*-GFP+ progenitor cells contribute to Tbr2+/NeuroD+ (arrows in **D1,D2**), Prox1+ GNPs (arrows in **D3,D4**) at P3. Schematic drawing of the expression patterns of *gfap*-GFP and transcription factors during the differentiation of GNPs **(E)**. Scale bars; 200 μm in **(A1–A4, B1–B4, C1–C3, D1,D3)**; 50 μm in **(D2,D4)**.

*Gfap*-GFP+/Sox2+ progenitors appear at E14 around the dentate notch (DN), and they migrate out of the ventricular zone (VZ) (Figure 1A1). Subsequently, they express an intermediate progenitor marker Tbr2 with some overlap (Figure 1A2). They then express the neuronal differentiation marker NeuroD (Figure 1A3), and Prox1, a marker for dentate granule neurons during the embryonic period (Figure 1A4). Similarly, *gfap*-GFP+/Sox2+ GNPs, *gfap*-GFP+/Tbr2+ GNPs, *gfap*-GFP+/NeuroD+ GNPs, and *gfap*-GFP+/Prox1+ GNPs were found at E16-17 and P3 (Figures 1B1–B4,D1–D4). In addition, some Sox2+/Tbr2+, Tbr2+/NeuroD+, and Tbr2+/Prox1+ GNPs were also found (Figures 1C1–C3). These data indicate that *gfap*-GFP+ GNPs sequentially express the transcription factors Sox2, Tbr2, NeuroD, and Prox1 during their differentiation into granule neurons in both the embryonic and early postnatal mouse dentate gyrus (DG).

### Zeb1 expression in Sox2+, Sox2+/Tbr2+, and Tbr2+/NeuroD+/Prox1+ GNPs

**Figure 2:**
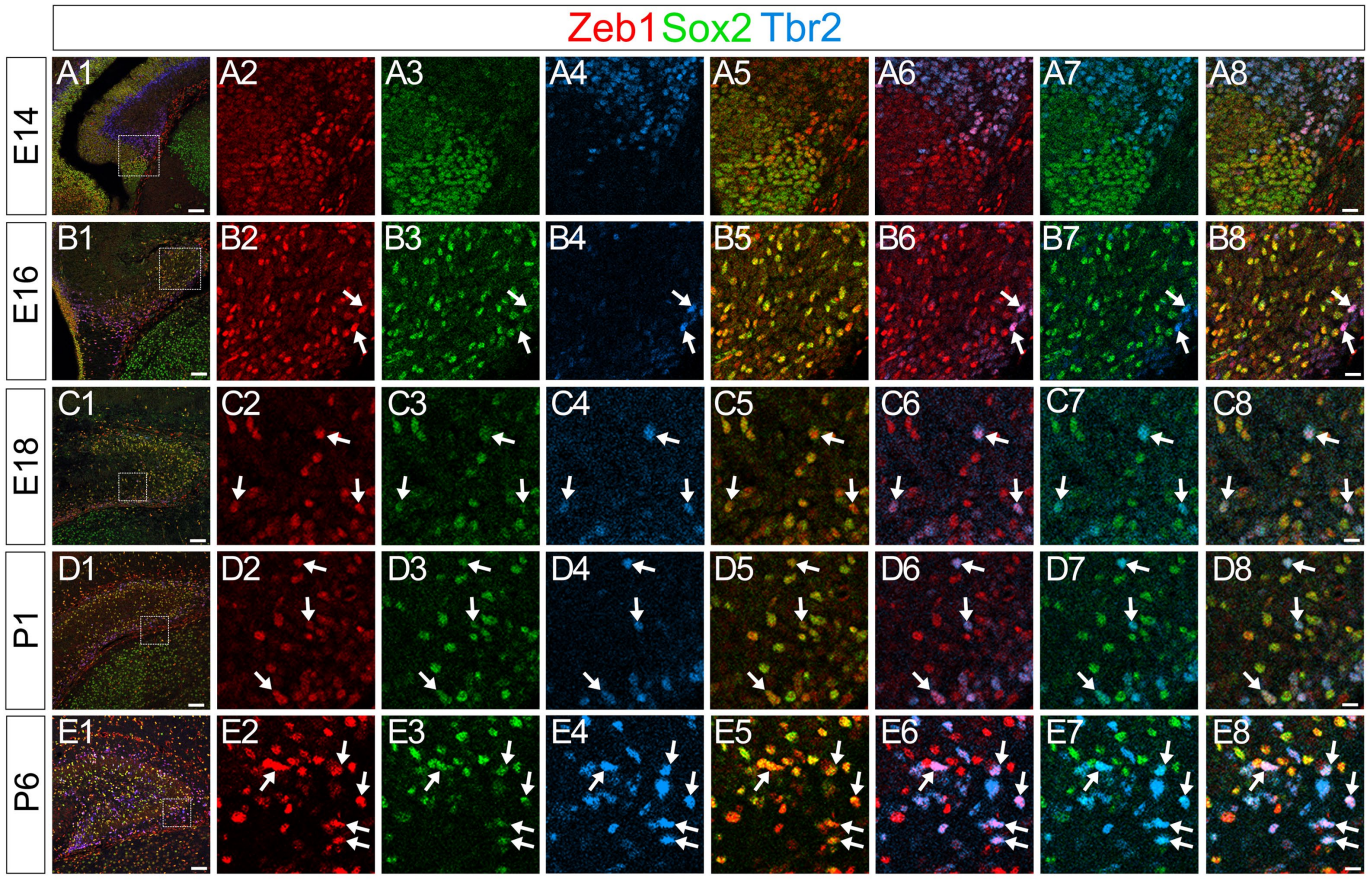
Zeb1 is expressed in Sox2+/Tbr2+ GNPs at E14-P6. Zeb1 is expressed in Sox2+/Tbr2+ GNPs at E14 (arrows in **A1–A8**), E16 (arrows in **B1–B8**), E18 (arrows in **C1–C8**), P1 (arrows in **D1–D8**) and P6 (arrows in **E1–E8**). The box in panels **(A1,B1,C1,D1,E1)** indicates the region shown in panels **(A2–A8,B2–B8,C2–C8,D2–D8,E2–E8)**. Scale bars; 200 μm in **(A1,B1,C1,D1,E1)**; 50 μm in **(A2–A8,B2–B8,C2–C8,D2–D8,E2–E8)**.

**Figure 3:**
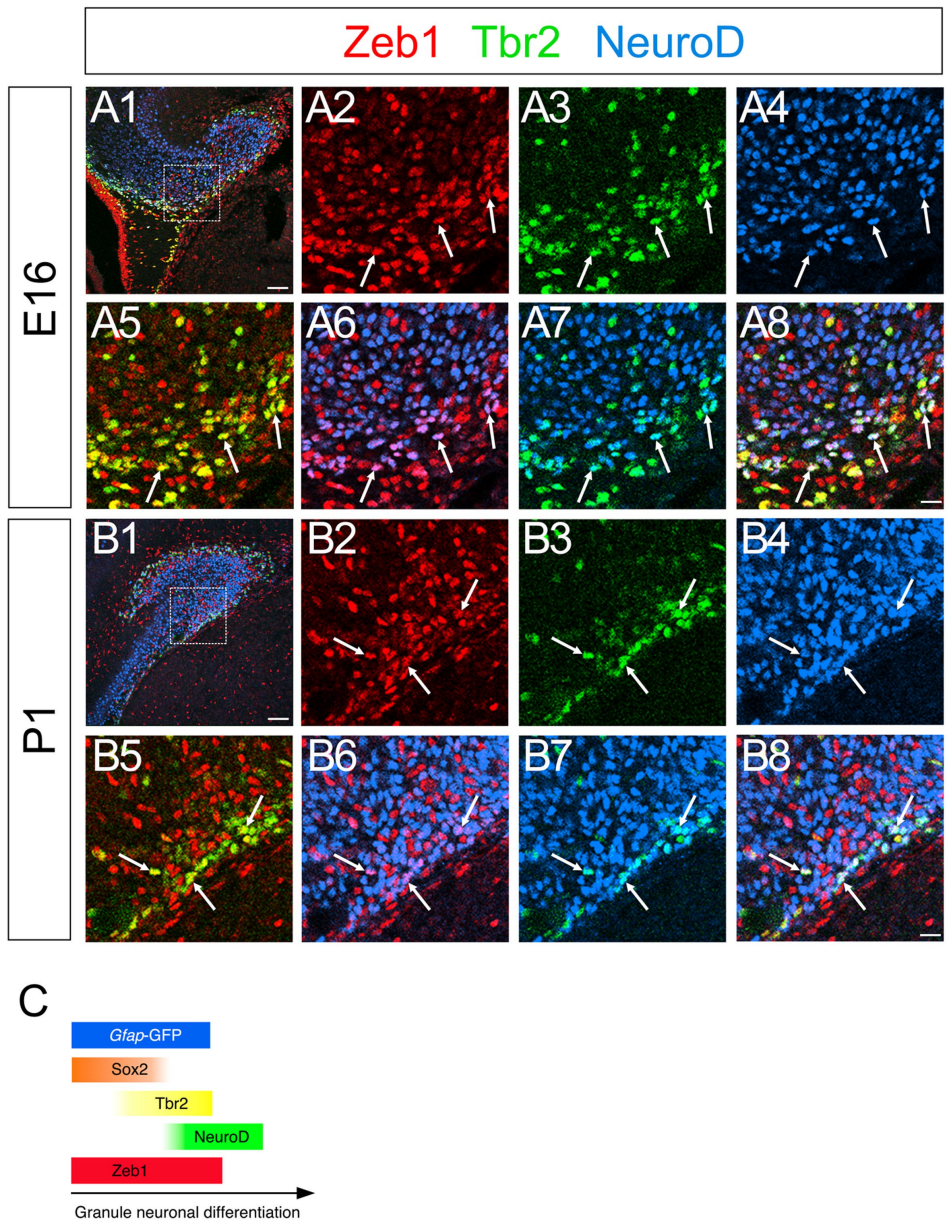
Zeb1 is expressed in both Tbr2+ and NeuroD+ GNPs. Zeb1 is expressed in Tbr2+ GNPs (yellow in **A8,B8**) and Tbr2+/NeuroD+ GNPs (white in **A8,B8**) at E16 **(A1–A8)** and P1 **(B1–B8)**. Box in panels **(A1,B1)** indicates region shown in panels **(A2–A8,B2–B8)**. Schematic drawing of the expression patterns of *gfap*-GFP, Zeb1 and other transcription factors during the differentiation of GNPs **(C)**. Scale bars; 200 μm in **(A1,B1)**; 50 μm in **(A2–A8,B2–B8)**.

**Figure 4:**
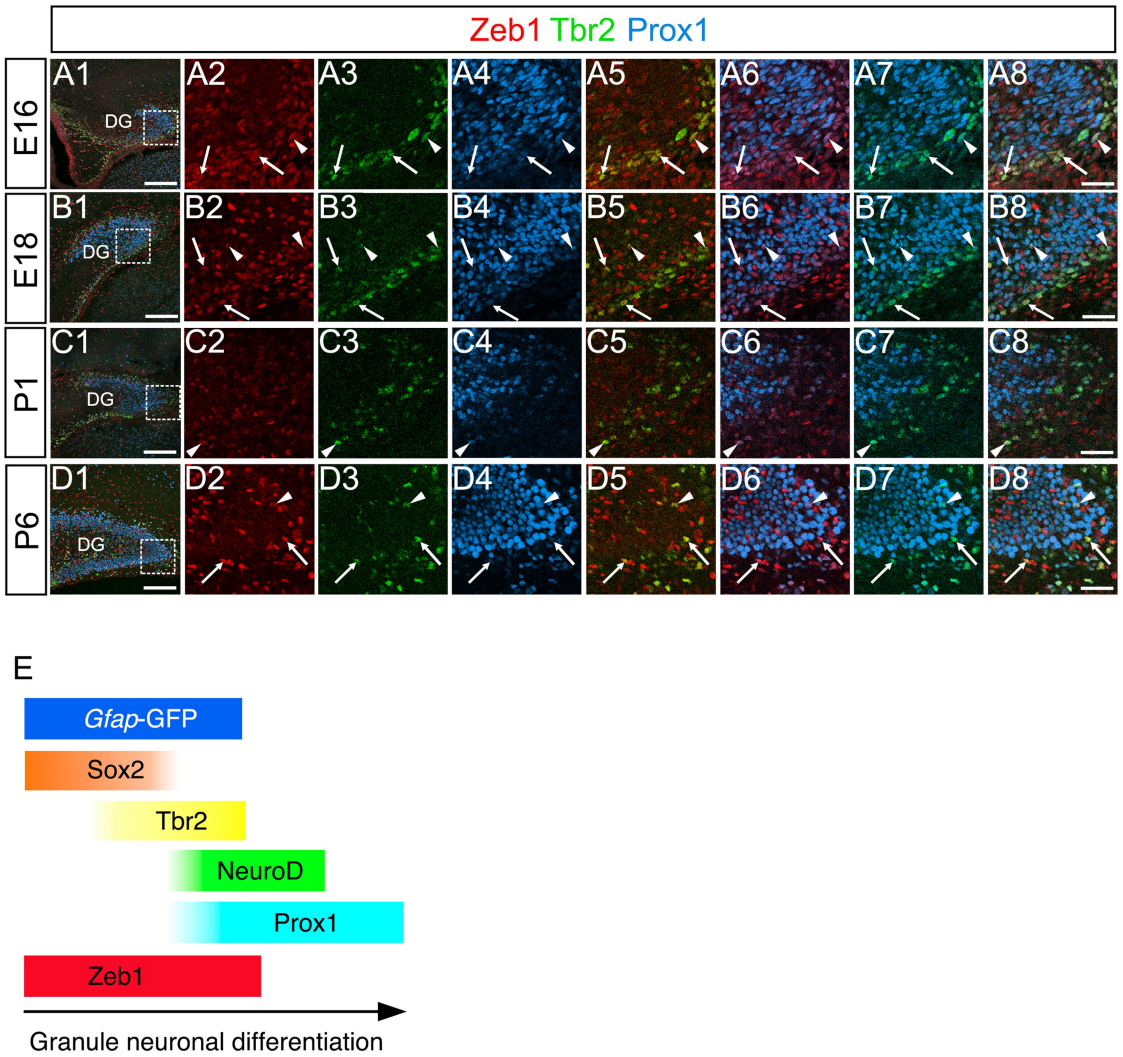
Zeb1 is expressed in both Tbr2+ and Tbr2+/Prox1+ GNPs in the developing mouse DG. Zeb1 is expressed in Tbr2+ GNPs (arrows in **A5,B5,C5,D5**) and Tbr2+/Prox1+ GNPs (arrowheads in **A8,B8,C8,D8**) at E16 **(A1–A8)**, E18 **(B1–B8)**, P1 **(C1–C8)**, and P6 **(D1–D8)**. The box in panels **(A1,B1,C1,D1)** indicates the region shown in panels **(A2–A8,B2–B8,C2–C8,D2–D8)**. Schematic drawing of the expression patterns of *gfap*-GFP, Zeb1 and other transcription factors during the differentiation of GNPs **(E)**. Note that at E17, Zeb1+ cells are composed of both early GNPs and Tbr2+ neuron-committed GNPs, whereas at P3, Zeb1+ cells are mainly Sox2+/Tbr2− early GNPs (see Supplementary Figure S1). Scale bars; 200 μm in **(A1,B1,C1,D1)**; 50 μm in **(A2–A8,B2–B8,C2–C8,D2–D8)**.

**Figure 5:**
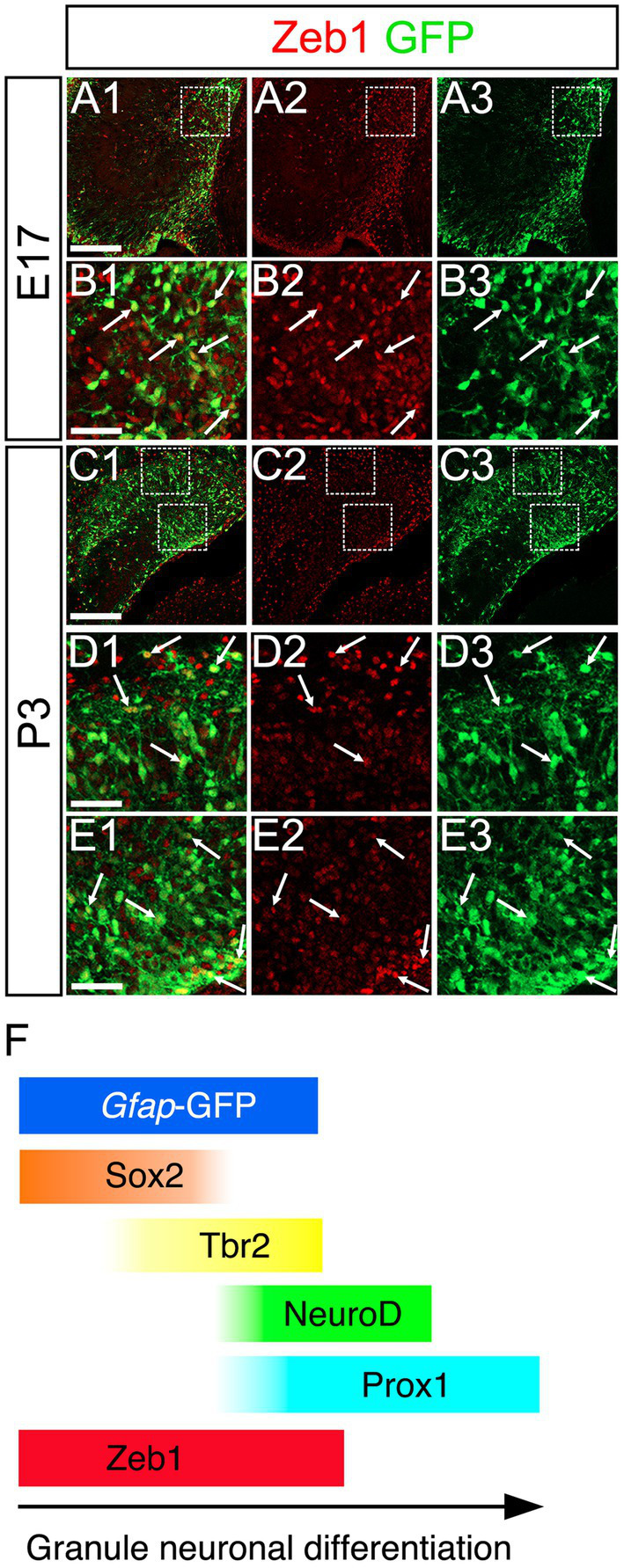
Zeb1 is expressed in *gfap*-GFP+ cells containing GNPs. Zeb1 is expressed in *gfap*-GFP+ cells at E17 (arrows in **B1–B3**) and P3 (arrows in **D1–D3**,**E1–E3**). The box in panels **(A1–A3,C1–C3)** indicates the region shown in panels **(B1–B3,D1–D3,E1–E3)**. Schematic drawing of the expression patterns of *gfap*-GFP, Zeb1 and other transcription factors during the differentiation of GNPs **(F)**. Note that at E17, Zeb1+ cells are composed of both early GNPs and Tbr2+ neuron-committed GNPs, whereas at P3, Zeb1+ cells are mainly Sox2+/Tbr2- early GNPs (see Supplementary Figure S1). Scale bars; 200 μm in **(A1–A3,C1–C3)**; 50 μm in **(B1–B3,D1–D3,E1–E3)**.

Consistent with previous studies that an EMT-TF Zeb1 controls the stemness of neural stem cells as well as cancer initiating cells (Ansieau et al., 2008; Chaffer et al., 2013; Gupta et al., 2021; Sabourin et al., 2009), Zeb1 expression was found in many Sox2+ neural stem/progenitors in the VZ of DG at E14 (Figures 2A1–A8). Co-expression of Zeb1 with Sox2 and Tbr2 was also found in the dentate migratory stream at E16 (Figures 2B1–B8). Similarly, at E18-P6, Zeb1 was expressed in Sox2+ and Sox2+/Tbr2+ progenitors in the DG (Figures 2C1–C8,D1–D8, E1–E8). Our data also showed that Zeb1+/Tbr2+ cells near the pial surface co-expressed NeuroD and Prox1 (Figures 3A1–A8,B1–B8; Figures 4A1–A8,B1–B8,C1–C8,D1–D8). These data suggest that Zeb1+ cells give rise, at least in part, to dentate granule neurons. Consistent with GFAP+ dentate progenitors and postnatal GFAP+ radial glia-like (RGL) cells giving rise to GNPs (Seki et al., 2014; Matsue et al., 2017), Zeb1 expression was found in *gfap*-GFP+ RGL cells at E17-P6 (Figures 5A1–A3,B1–B3,C1–C3,D1–D3,E1–E3,F). Taken together, Zeb1 is expressed in GNPs at different stages of differentiation: predominantly in Sox2+ progenitors (Supplementary Figure S1A, 49.5% at E17, 56.4% at P3), to a lesser extent in Tbr2+ early GNPs (Supplementary Figure S1B, 44.1% at E17, 29.5% at P3) and Tbr2+/NeuroD+/Prox1+ neuron-committed GNPs (Figures 2–4).

Taken together, Zeb1 is expressed in GNPs at different stages of differentiation: predominantly in Sox2+ progenitors (Supplementary Figure S1A, 49.5% at E17, 56.4% at P3), to a lesser extent in Tbr2+ early GNPs (Supplementary Figure S1B, 44.1% at E17, 29.5% at P3) and Tbr2+/NeuroD+/Prox1+ neuron-committed GNPs (Figures 2–4). The ratio of Zeb1+/Sox2+ cells among Zeb1+ cells remained relatively stable, whereas the ratio of Zeb1+/Tbr2+ cells among Zeb1 + cells was significantly decreased. Thus, at E17, Zeb1+ cells are composed of both early GNPs and Tbr2+ neuron-committed GNPs, whereas at P3, Zeb1+ cells are mainly Sox2+/Tbr2− early GNPs.

### Scrt2 is expressed in *gfap*-GFP+/Tbr2+ GNPs and Prox1+ early differentiating granule neurons but not in Cajal–Retzius neurons

**Figure 6:**
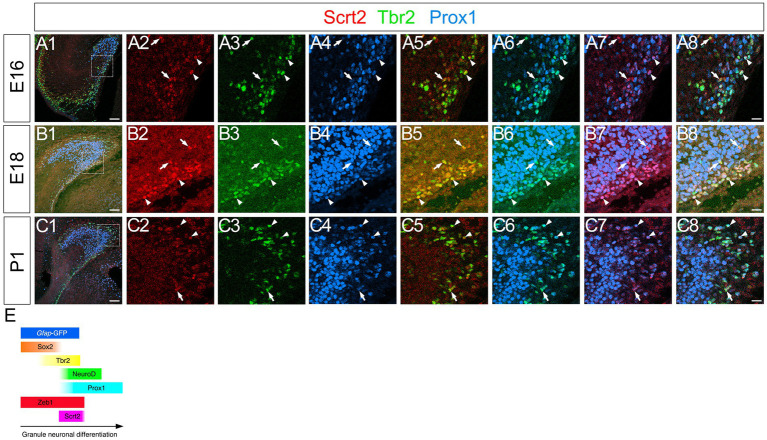
Scrt2 is expressed in Tbr2+ GNPs and Tbr2+/Prox1+ GNPs at E16, E18, and P1. Scrt2 is expressed in Tbr2+ and Tbr2+/Prox1+ GNPs at E16, E18, and P1 (arrows in **A5,B5,C5** and arrowheads in **A8,B8,C8**, respectively). The box in panels **(A1,B1,C1)** indicates the region shown in panels **(A2–A8, B2–B8, C2–C8)**. Schematic drawing of the expression patterns of *gfap*-GFP, Zeb1, Scrt2 and other transcription factors during the differentiation of GNPs **(E)**. Note that at E17, Zeb1+ cells are composed of both early GNPs and Tbr2+ neuron-committed GNPs, whereas at P3, Zeb1+ cells are mainly Sox2+/Tbr2- early GNPs (see Supplementary Figure S1). Scale bars; 200 μm in **(A1,B1,C1)**; 50 μm in **(A2–A8, B2–B8, C2–C8)**.

Scrt2 has been shown to be expressed at the onset of neuronal migration in the cerebral cortex (Itoh et al., 2013). This led us to examine whether Scrt2 is expressed in migrating DG progenitors. At E14-P6 Scrt2 expression was found in the cells migrating away from the VZ, and they are aligned in the subpial and hilar regions, corresponding to the prospective GCL and ML, respectively (Figures 6A1–A8,B1–B8,C1–C8; Supplementary Figures S2A1–A6,B1–B6,C1–C6). The vast majority of Scrt2+ cells were Tbr2+ (Supplementary Figure S1C, 78.7% at E16, 65.4% at P3, 74.8% at P6), Prox1+ (Supplementary Figure S1D, 61.9% at E16, 50.1% at P6) GNPs at E16-P6. Consistent with *gfap*-GFP+ progenitors generating GNPs and subsequently granule neurons in the DG (Seki et al., 2014), Scrt2 expression was also found in some *gfap*-GFP+ progenitors (Supplementary Figure S2). These data suggest that Scrt2 is mainly expressed in Tbr2+ GNPs derived from *gfap*-GFP+ progenitors.

**Figure 7:**
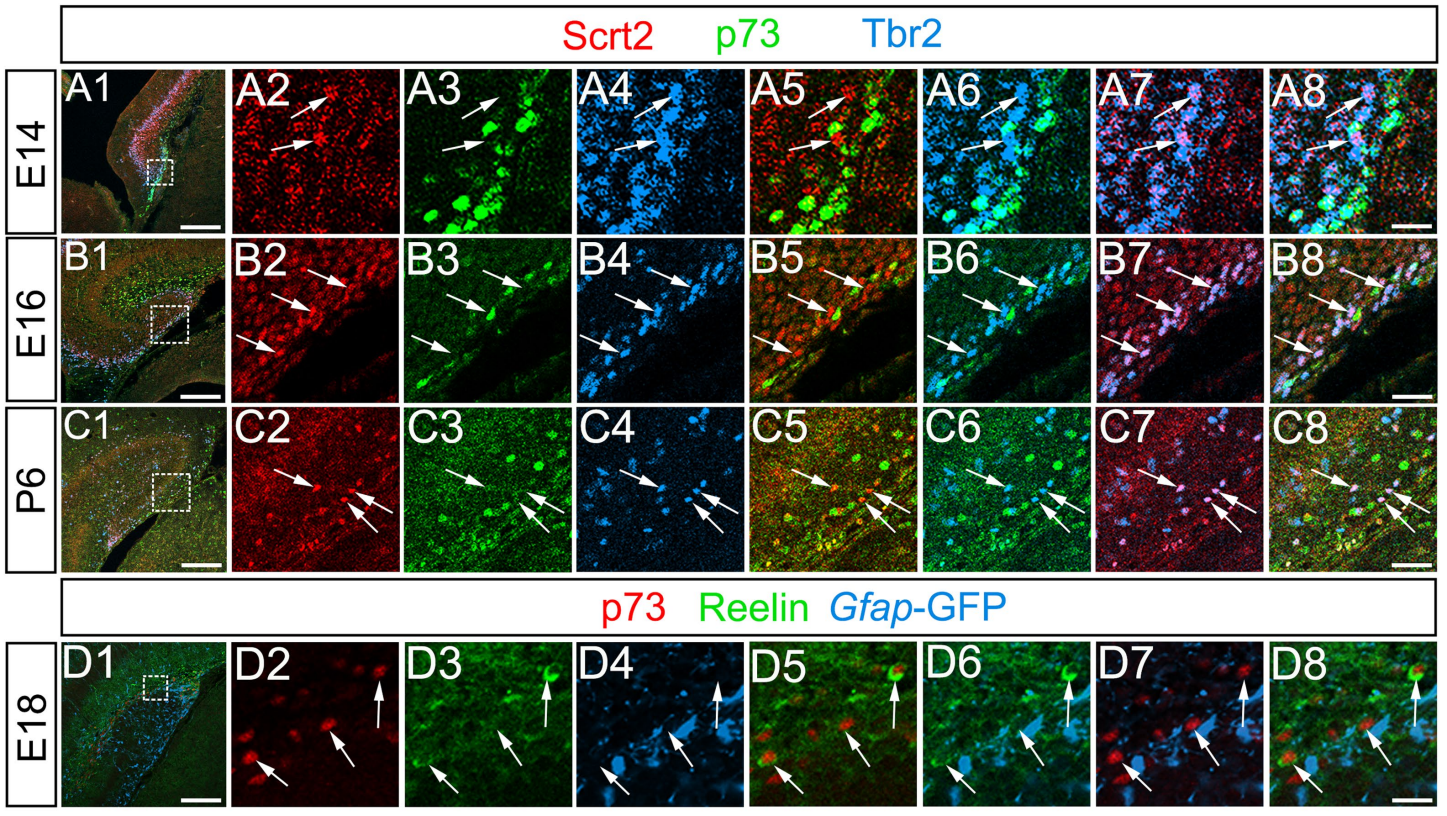
Scrt2 is expressed in Tbr2+ GNPs but not in p73+/Reelin+ Cajal–Retzius neurons. Scrt2 is expressed in Tbr2+ GNPs but not in p73+ cells at E14, E16, and P6 (arrows in A7, B7, C7, respectively). P73 is expressed in reelin+ Cajal–Retzius neurons but not in *gfap*-GFP+ cells at E18 (arrows in **D1–D8**). The box in panels **(A1,B1,C1,D1)** indicates the region shown in panels **(A2–A8,B2–B8,C2–C8,D2–D8)**. Scale bars; 200 μm in **(A1,B1,C1,D1)**; 50 μm in **(A2–A8,B2–B8,C2–C8,D2–D8)**.

Previous studies have shown that Tbr2+ progenitors differentiate not only into dentate granule neurons, but also into reelin+ Cajal–Retzius (CR) neurons at the hippocampal fissure (Hodge et al., 2013). This raised the question of whether Scrt2 is also expressed in a Tbr2+ CR neuronal lineage at the hippocampal fissure. However, Scrt2 and p73 expression did not overlap, while some p73+/Tbr2+ cells were found at the hippocampal fissure (Figures 7A1–A8,B1–B8,C1–C8; Supplementary Figure S4, data not shown). In addition, Scrt2 was not expressed in reelin+ Cajal–Retzius (CR) neurons (data not shown). Our data also showed that reelin+ CR neurons at the hippocampal fissure did not express *gfap*-GFP (Figures 7D1–D8). Thus, neither *gfap*-GFP+ cells nor Scrt2+ progenitors contribute to CR neurons. Consistent with this, both p73 and Lhx1/5, markers of CR neuronal progenitors were not expressed in *gfap*-GFP+ progenitors (Figures 7D1–D8, data not shown). These data indicate that Scrt2+/Tbr2+ cells are GNPs but not CR neuronal progenitors.

### Scrt2 is also expressed in some Zeb1+/Sox2+ GNPs

**Figure 8:**
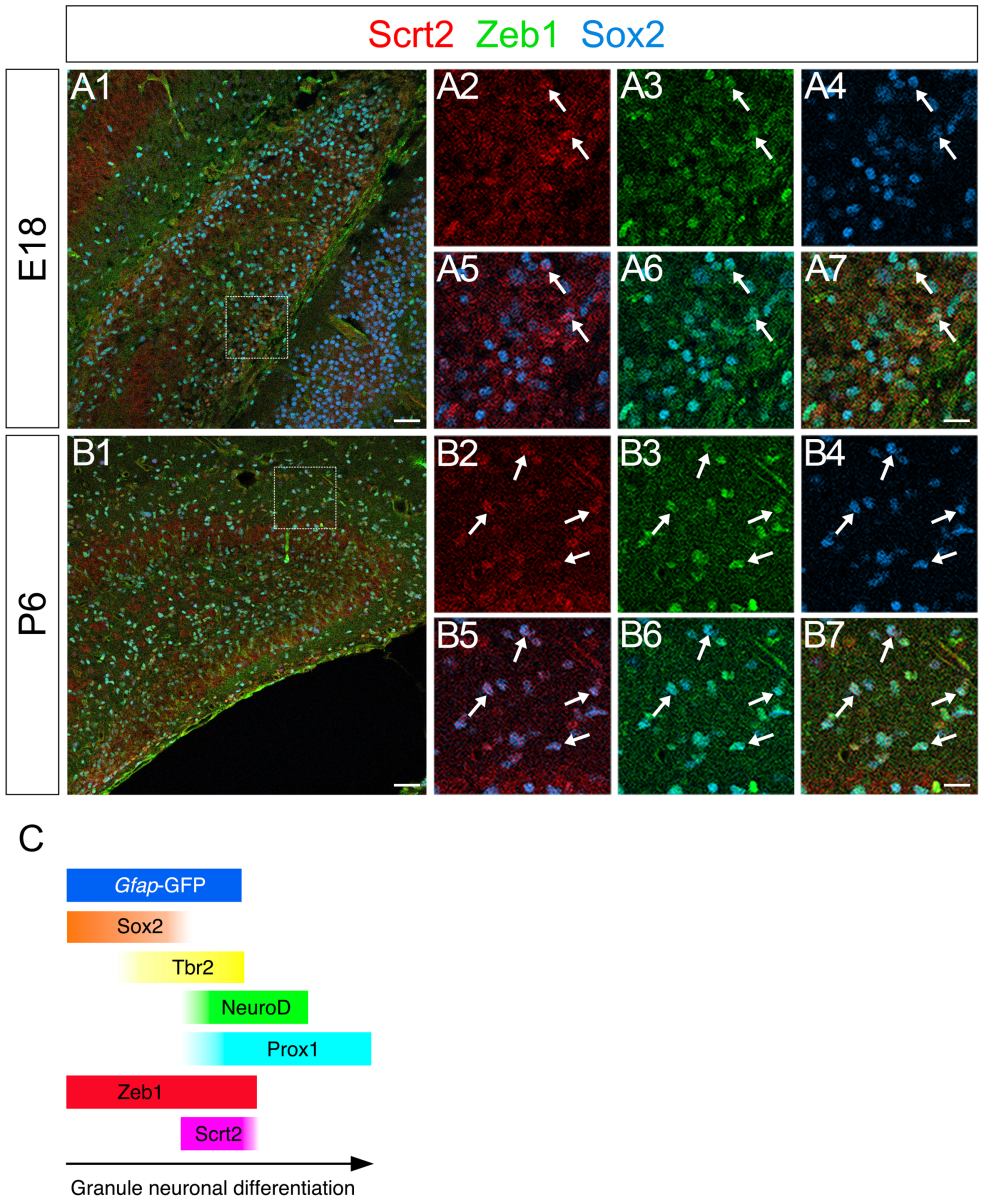
Scrt2 is expressed in Zeb1 + Sox2+ GNPs in the DG at E18 and P6. Scrt2 is expressed in Zeb1+/Sox2+ GNPs at E18 (**A1–A7**, arrows in **A2–A7**) and P6 (**B1–B7**, arrows in **B2–B7**). **(A2–A7)** are higher magnification of boxed area in **(A1)**. **(B2–B7)** are higher magnification of the boxed area in **(B1)**. Box in panels **(A1,B1)** indicates region shown in panels **(A2–A7,B2–B7)**. Schematic drawing of the expression patterns of *gfap*-GFP, Zeb1, Scrt2 and other transcription factors during the differentiation of GNPs **(C)**. Note that at E17, Zeb1+ cells are composed of both early GNPs and Tbr2+ neuron-committed GNPs, whereas at P3, Zeb1+ cells are mainly Sox2+/Tbr2− early GNPs (see Supplementary Figure S1). Scale bars; 200 μm in **(A1,B1)**; 50 μm in **(A2–A7,B2–B7)**.

The vast majority of *gfap*-GFP+ cells express the neural stem/progenitor cell marker Sox2 both in the VZ and in the parenchyma of the developing DG (Figure 1). While some of these differentiate into Prox1+ dentate granule neurons (Figure 1), many Sox2+/*gfap*-GFP+ RGL progenitors were retained (Figure 1). Given our data that Scrt2 is expressed in some *gfap*-GFP+ cells (Supplementary Figure S3), this raised the possibility that Scrt2 is co-expressed with Zeb1 in Sox2+ progenitors. Indeed, co-expression of Scrt2 and Zeb1 was found in Sox2_low_ + at E18 and P6 (Figures 8A1–A7,B1–B7).

### Nkx6-2 expression in Tbr2+/Prox1+ GNPs and Prox1+ differentiating granule neurons in postnatal but not embryonic GNPs

**Figure 9:**
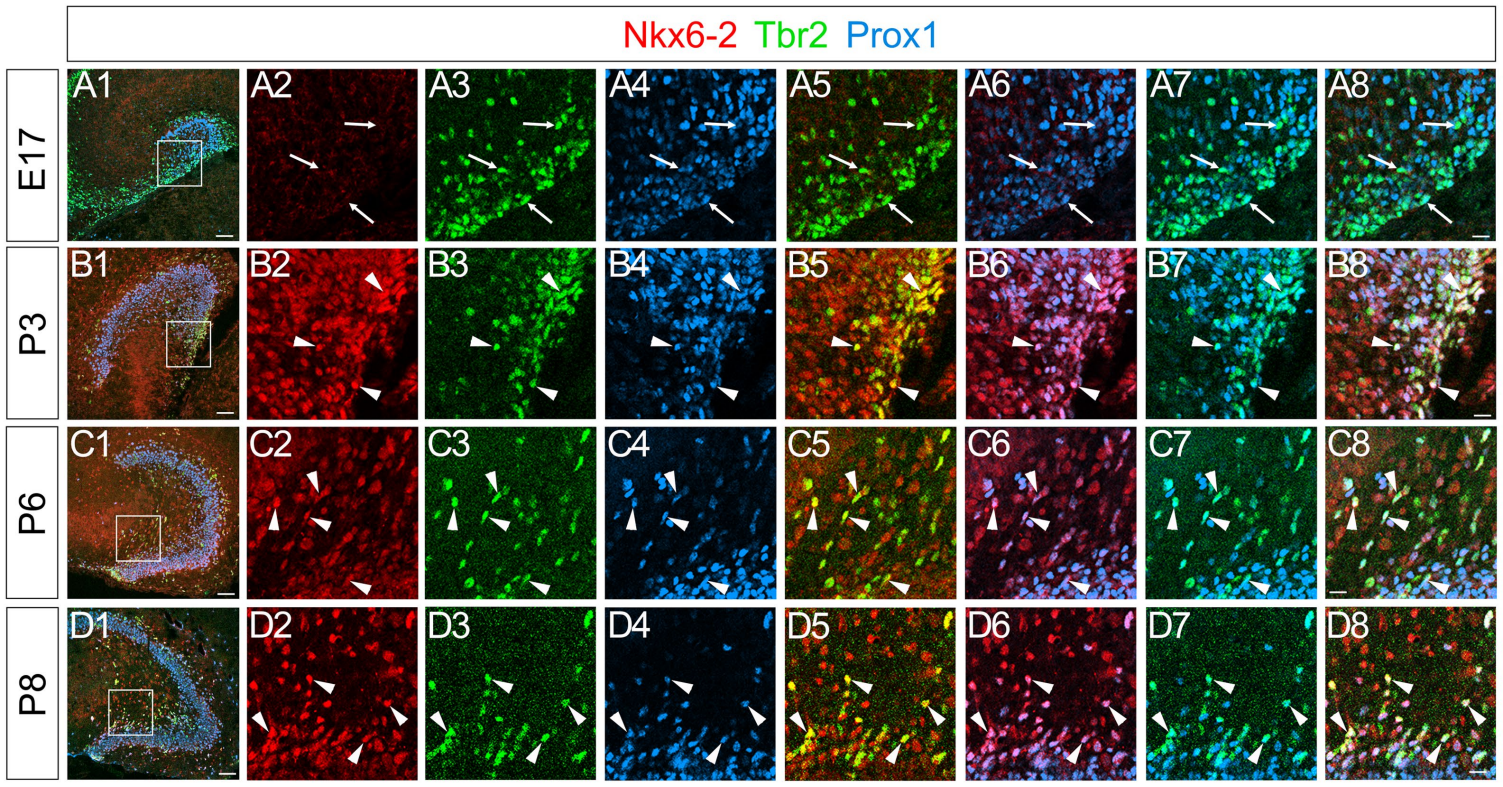
Nkx6-2 is expressed in Tbr2+/Prox1+ GNPs in the DG during the postnatal period, but not during the embryonic period. No obvious Nkx6-2 expression was observed in the DG at E17 **(A2)**, whereas Tbr2+/Prox1+ GNPs were found (arrows in **A3–A8**). In contrast, Nkx6-2 is expressed in Tbr2+/Prox1+ GNPs at P3, P6, and P8 (arrowheads in **B1–B8,C1–C8,D1–D8**, respectively). The box in panels **(A1,B1,C1,D1)** indicates the region shown in panels **(A2–A8,B2–B8,C2–C8,D2–D8)**. Scale bars; 200 μm in **(A1,B1,C1,D1)**; 50 μm in **(A2–A8,B2–B8,C2–C8,D2–D8)**.

A recent meta-analysis of EMT datasets revealed that there are five distinct types of EMT, including stemness EMT and neuronal EMT (Liang et al., 2016). A transcription factor Nkx6-2 was identified to be involved in neuronal specification and classified as a neuronal EMT-TF. We then examined the expression pattern of Nkx6-2 in the developing DG. Intriguingly, while Nkx6.2 expression was not found in the developing DG at E17 (Figures 9A1–A8), it was expressed in Tbr2+/Prox1+ GNPs and Prox1+ early differentiating granule neurons (69 ± 5%) at P3-P8 (Figures 9B1–B8,C1–C8,D1–D8). Consistent with this, Nkx6-2 mRNA expression was found in postnatal but not embryonic DG (Allen Brain Atlas, https://developingmouse.brainmap.org/search/show?page_num=0&page_size=3&no_paging=false&exact_match=true&search_term=Nkx6-2&search_type=gene). These data suggest that Nkx6.2 expression is somehow restricted to postnatal GNPs and early differentiating granule neurons.

### Expression of Zeb1, Scrt2, and Nkx6-2 in postnatal GNPs

**Figure 10:**
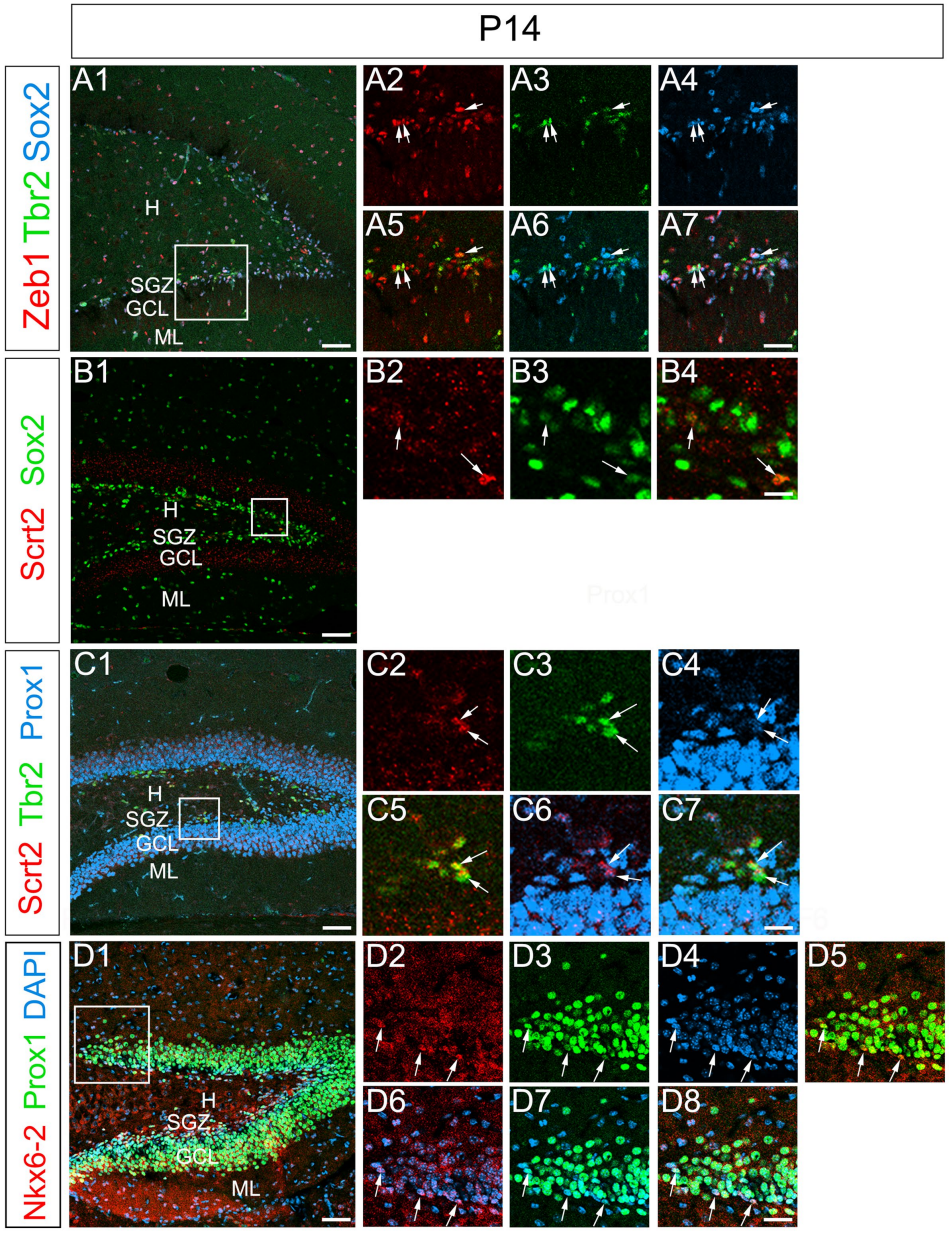
Preserved expression patterns of Zeb1, Scrt2, and Nkx6-2 in the DG at P14. Zeb1 is expressed in Sox2+/Tbr2+ GNPs at P14 (arrows in **A1–A7**). Scrt2 is expressed in Sox2_low_ + GNPs **(B1–B7)**. Scrt2 is expressed in Tbr2+/Prox1+ GNPs. Box in panels **(A1,B1,C1,D1)** indicates region shown in panels **(A2–A7,B2–B4,C2–C7,D2–D8)**. Scale bars; 200 μm in **(A1,B1,C1,D1)**; 50 μm in **(A2–A7,B2–B4,C2–C7,D2–D8)**.

The formation of granule cell layer of the DG is almost complete at P14 (Figure 10). We examined whether the temporal expression profiles of Zeb1 and Scrt2 were maintained in the DG at P14. Zeb1 was found to be co-expressed with Sox2 and Tbr2 in the SGZ cells (Figures 10A1–A7). Scrt2 expression was observed in Sox2_low_+, Tbr2+, Prox1+ cells at the SGZ, but not in Prox1 + GCL (Figures 10B1–B4, C1–C7). Overall, Zeb1 is expressed predominantly in Sox2+ RGL cells (Figures 10A1–A7). Scrt2 is mainly expressed in Tbr2+ GNPs (Figures 10C1–C7). Our data also show that Nkx6-2 is expressed in the SGZ where Prox1+ GNPs are located (Figures 10D1–D8).

## Discussion

Our results reveal distinct spatio-temporal expression profiles of the EMT-TFs factors Zeb1, Scrt2, and Nkx6-2 during GNP differentiation in the developing mouse DG. We show that Zeb1 and Scrt2 exhibit sequential but partially overlapping expression in embryonic and postnatal GNP populations, whereas Nkx6-2 is selectively expressed in postnatal GNPs. These results highlight temporally coordinated transcriptional programs that control GNP developmental trajectories.

### Zeb1 and Scrt2 expression dynamics define distinct GNP differentiation states

Zeb1 is predominantly expressed in *gfap*-GFP++/Sox2+ neural stem/progenitor cells and radial glia-like (RGL) progenitors (Figures 2–4), with progressively decreasing expression in Tbr2+ intermediate GNPs and Tbr2+/Prox1+/NeuroD+ neuron-committed GNPs (Figures 2–4). In contrast, Scrt2 shows robust expression in Tbr2+/Prox1+/NeuroD+ GNPs and Prox1+ early differentiating granule neurons (Figures 4, 6, 8). This complementary expression pattern suggests that Zeb1 and Scrt2 mark sequential stages of GNP differentiation, from stem/progenitor pools through intermediate and neuron-committed progenitor states.

### Distinct roles for Zeb1 and Scrt2 in regulating GNP stemness and differentiation

Zeb1 and Sox2 are interdependently involved in the regulation of stemness. Zeb1 inhibits stemness-repressing miRNAs such as miR-200 that target Sox2, thereby maintaining Sox2 expression and stemness (Burk et al., 2008; Singh et al., 2017). In contrast, Zeb1 knockdown decreases the expression level of Sox2 (Pérez et al., 2021). It was also shown that Sox2 can directly bind to the promoter region of Zeb1, indicating a feedback loop (Singh et al., 2017). These data suggest that Zeb1 and Sox2 expressions are mutually dependent.

Regarding Scrt2, its expression in Tbr2+ intermediate progenitors is consistent with its known role in promoting neuronal differentiation. Scrt2 has been shown to regulate the cell cycle exit of neural progenitors by inhibiting cyclin D1 expression and promoting p27 expression (Rodríguez-Aznar and Nieto, 2011). This function may facilitate the transition of Tbr2+ intermediate progenitors to NeuroD1+ neuroblasts in the DG.

The postnatal-specific expression of Nkx6-2 in Tbr2+/Prox1+ cells suggests a potential role in regulating the transition from intermediate progenitors to immature granule neurons. While direct interactions between Nkx6-2 and these differentiation markers have not been extensively studied in the DG, Nkx6-2 has been shown to promote neural migration in other contexts (Toch et al., 2020). Its expression pattern in our study suggests that it may play a similar role in granule neuron development.

The enrichment of Zeb1 in GFAP+/Sox2+ stem/progenitor populations is also consistent with its reported role in inhibiting cellular senescence and maintaining neural progenitor proliferation (Liu et al., 2008; Singh et al., 2016). Zeb1 may repress pro-apoptotic genes such as p73 to maintain the undifferentiated state of dentate GNP-producing progenitors (Bui et al., 2009). In addition, by downregulating cell polarity genes, Zeb1 prevents premature epithelial-mesenchymal transition (EMT) and preserves the stem/progenitor properties of GNP-generating cells (Singh et al., 2016).

In contrast, Scrt2 expression in Tbr2+/ Prox1+ neuron-committed GNPs and Prox1+ differentiating granule neurons (Figures 4, 6, 8) suggests a role in promoting neuronal differentiation. Consistent with reported functions in the neocortex and spinal cord (Itoh et al., 2013; Rodríguez-Aznar et al., 2013), Scrt2 may facilitate neuronal migration and cell cycle exit of dentate GNPs. The restricted expression in the granule lineage, but not in Cajal–Retzius precursors (Figure 7), suggests a lineage-specific role in regulating GNP differentiation.

### Distinct transcriptional regulation of embryonic versus postnatal GNPs

A striking difference between embryonic and postnatal stages is the selective expression of the neuronal EMT regulator Nkx6-2 in postnatal Tbr2+/Prox1+ GNPs (Figures 9B1–D8). This temporal specificity suggests divergent transcriptional programs governing the dynamics of GNP differentiation in embryonic versus postnatal neurogenic niches. While embryonic GNPs rely on Zeb1 and Scrt2, postnatal GNPs may use Nkx6-2 to facilitate neuronal differentiation through EMT-associated mechanisms.

Recent research has highlighted a transition from radial glia/neural stem cells to radial glial-like cells (RGLs) during postnatal development. For example, Matsue et al. (2017) demonstrated that *gfap*-GFP+ radial glia transition to *gfap*-GFP+/BLBP+ RGLs during the postnatal period. Consistent with this, recent single cell RNA-seq data from Hochgerner et al. (2018) revealed a shift in the molecular identity of radial glia after postnatal day 5, which persists into adult stages. In contrast, Hopx-expressing dentate progenitors, which give rise to adult RGLs, retain a consistent molecular signature throughout development (Berg et al., 2019). These data imply that RGLs are a heterogenous cell population. Our recent findings support this notion by showing that phospho-Smad3 is expressed in a subpopulation of RGLs throughout development and in adulthood.

In summary, our data have revealed that the temporally coordinated expression of EMT-TFs Zeb1 and Scrt2 in GNPs is maintained throughout development. In contrast, Nkx6-2 expression is restricted to postnatal GNPs, highlighting a previously unrecognized difference between embryonic and postnatal GNPs during DG development.

## Data Availability

The original contributions presented in the study are included in the article/Supplementary material, further inquiries can be directed to the corresponding author.

## Supplementary material

SUPPLEMENTARY Figure S1 Marker expression profiles of Zeb1+ and Scrt2+ GNPs. Zeb1 is expressed in Sox2+ progenitors at E17 (49.5%, 4 mice, 3 sections for each, 12 sections in total) and P3 (56.4%, 5 mice, 14 sections) **(A)**. No significant difference was found between E17 and P3. Two-tailed unpaired t test. *p* = 0.1824. Zeb1 is expressed in Tbr2+ progenitors at E17 (44.1%, 4 mice, 3 sections for each, 12 sections in total) and P3 (29.5%, 4 mice, 3 sections for each, 12 sections in total) **(B)**. Statistically significant difference was found between E17 and P3. Two-tailed unpaired *t* test. *p* = 0.0007. Scrt2 is expressed in Tbr2+ progenitors at E16 (78.7%, 4 mice, 3 sections for each, 12 sections in total), P3 (65.4%, 4 mice, 3 sections for each, 12 sections in total), and P6 (74.8%, 3 mice, 2 sections for each, 6 sections in total). No significant difference was found by one-way ANOVA among the developmental ages. Scrt2 is expressed in Prox1+ cells at E16 (61.9%, 4 mice, 2-3 sections for each, 11 sections in total) and P6 (50.1%, 3 mice, 2-3 sections for each, 7 sections in total). Statistically significant difference was found between E16 and P6. Two-tailed unpaired *t* test. *p* = 0.047.

SUPPLEMENTARY Figure S2 Scrt2 is co-expressed with Tbr2 in the DG at E14, P3, and P6. Scrt2 is expressed in Tbr2+ GNPs in the DG at E14, P3, and P6 (arrowheads in **A5,B5,C5**, respectively). The box in panels **(A1,B1,C1)** indicates the region shown in panels **(A2–A6,B2–B6,C2–C6)**. Scale bars; 200 mm in **(A1,B1,C1)**; 50 mm in **(A2–A6, B2–B6, C2–C6)**.

SUPPLEMENTARY Figure S3 Scrt2 expression in *gfap*-GFP+ DG progenitors. Scrt2 is expressed in *gfap*-GFP+ DG progenitors at E14, E17, and P1 (arrows in **A2–A4,B2–B4,C1–C4**, respectively). The box in panels **(A1,B1,C1)** indicates the region shown in panels **(A2–A4,B2–B4,C2–C4)**. Schematic drawing shows the expression patterns of *gfap-*GFP and Scrt2 during the differentiation of GNPs. Scale bars; 200 mm in **(A1,B1,C1)**; 50 mm in **(A2–A4,B2–B4,C2–C4)**.

SUPPLEMENTARY Figure S4 Scrt2 is not expressed in p73+ Cajal-Retzius neurons. Scrt2 is not expressed in p73+ Cajal-Retzius neurons at P1 and P6 (arrowheads in **A2–A5,B2–B5**, respectively). The box in panels **(A1,B1)** indicates the region shown in panels **(A2–A5,B2–B5)**. Scale bars; 200 mm in **(A1,B1)**; 50 mm in **(A2–A5,B2–B5)**.
