# Supplementary figures and images for: Differentiation stage-specific expression of transcriptional regulators for epithelial mesenchymal transition in dentate granule progenitors
Source: Front Neurosci. 2024 Aug 29;18:1425849. doi: 10.3389/fnins.2024.1425849 (PMC11390541; doi:10.3389/fnins.2024.1425849)

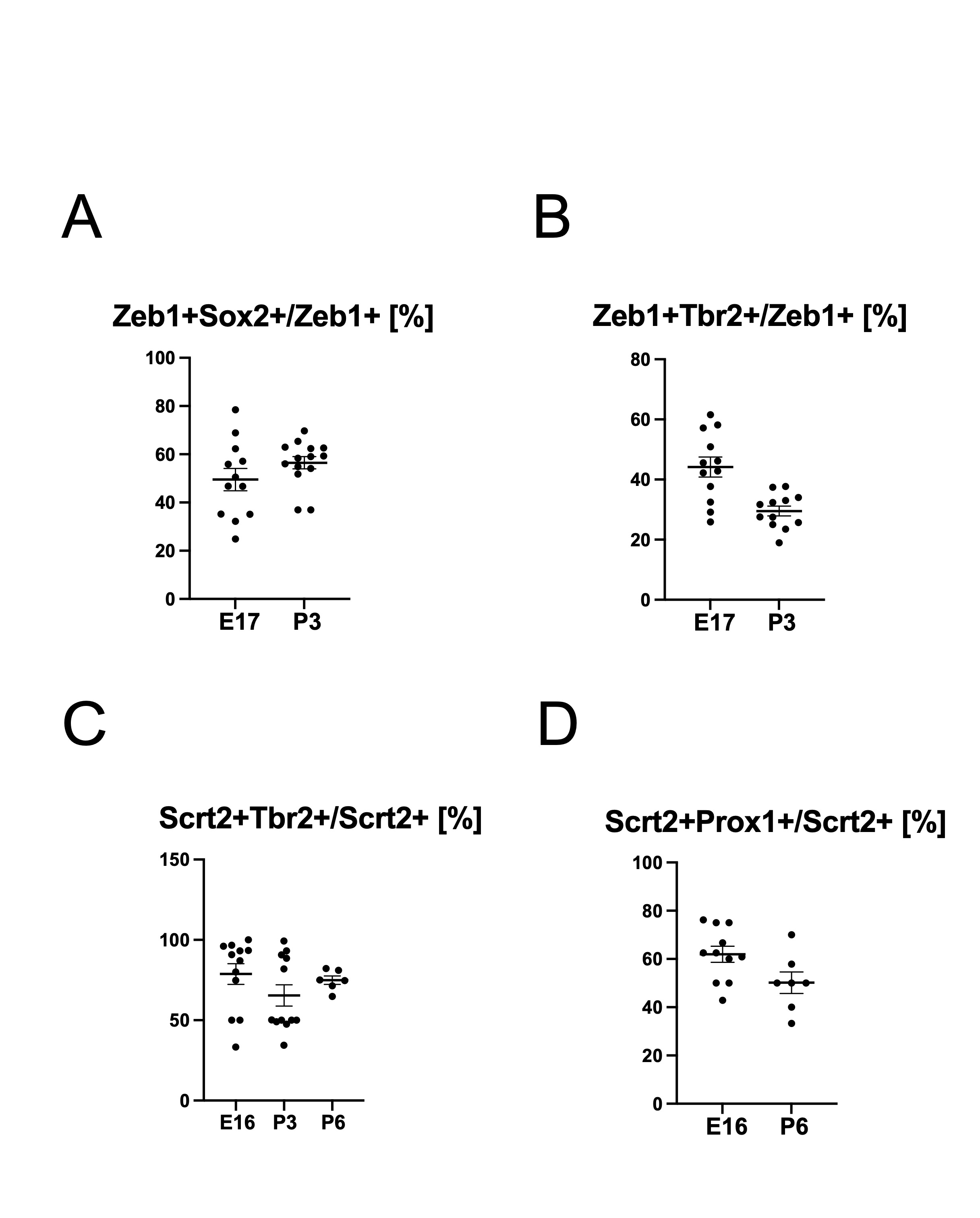

Supplement: SUPPLEMENTARY Figure S1 — Marker expression profiles of Zeb1+ and Scrt2+ GNPs. Zeb1 is expressed in Sox2+ progenitors at E17 (49.5%, 4 mice, 3 sections for each, 12 sections in total) and P3 (56.4%, 5 mice, 14 sections) (A). No significant difference was found between E17 and P3. Two-tailed unpaired t test. p = 0.1824. Zeb1 is expressed in Tbr2+ progenitors at E17 (44.1%, 4 mice, 3 sections for each, 12 sections in total) and P3 (29.5%, 4 mice, 3 sections for each, 12 sections in total) (B). Statistically significant difference was found between E17 and P3. Two-tailed unpaired t test. p = 0.0007. Scrt2 is expressed in Tbr2+ progenitors at E16 (78.7%, 4 mice, 3 sections for each, 12 sections in total), P3 (65.4%, 4 mice, 3 sections for each, 12 sections in total), and P6 (74.8%, 3 mice, 2 sections for each, 6 sections in total). No significant difference was found by one-way ANOVA among the developmental ages. Scrt2 is expressed in Prox1+ cells at E16 (61.9%, 4 mice, 2-3 sections for each, 11 sections in total) and P6 (50.1%, 3 mice, 2-3 sections for each, 7 sections in total). Statistically significant difference was found between E16 and P6. Two-tailed unpaired t test. p = 0.047. [file Image_1.JPEG]

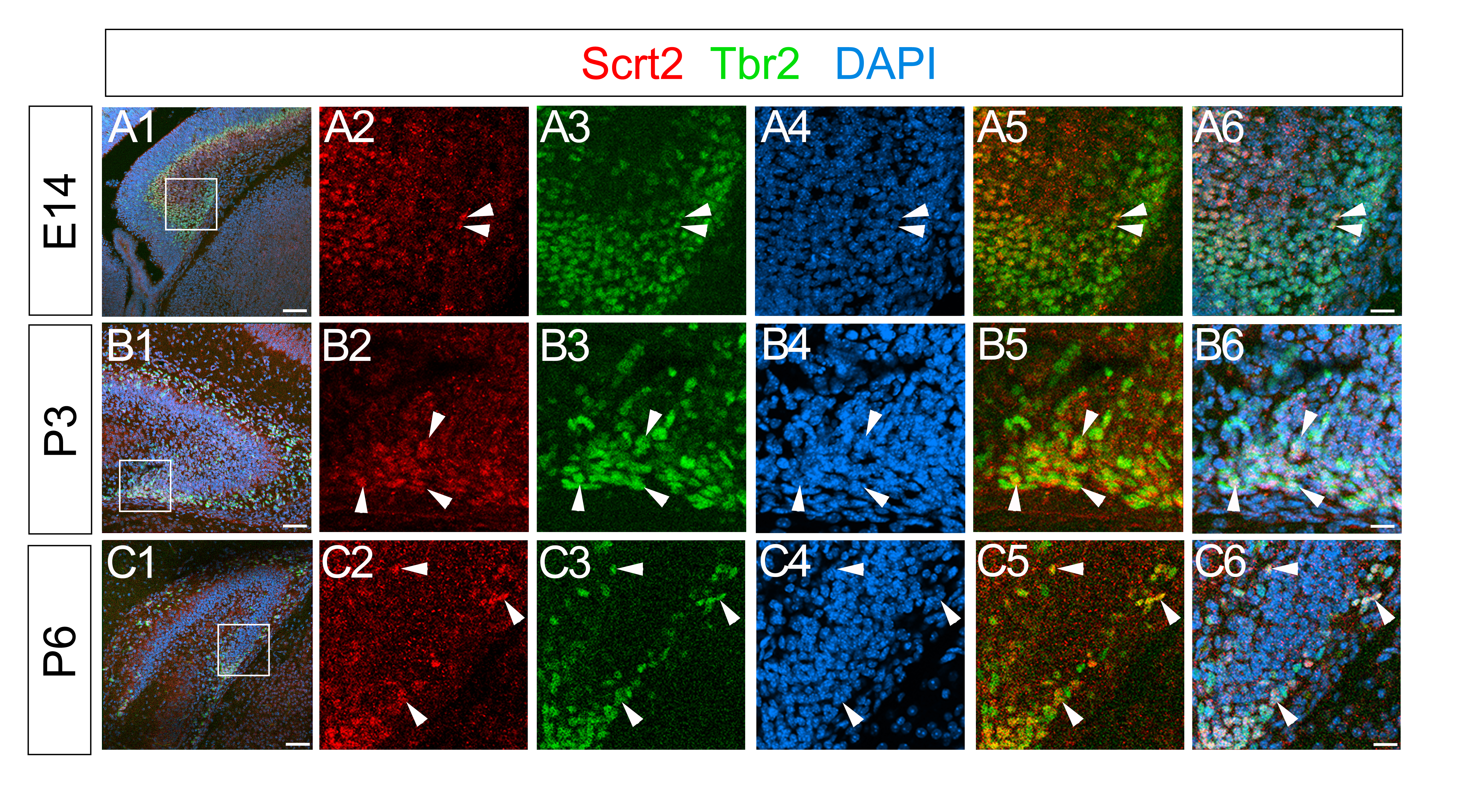

Supplement: SUPPLEMENTARY Figure S2 — Scrt2 is co-expressed with Tbr2 in the DG at E14, P3, and P6. Scrt2 is expressed in Tbr2+ GNPs in the DG at E14, P3, and P6 (arrowheads in A5,B5,C5, respectively). The box in panels (A1,B1,C1) indicates the region shown in panels (A2–A6,B2–B6,C2–C6). Scale bars; 200 mm in (A1,B1,C1); 50 mm in (A2–A6, B2–B6, C2–C6). [file Image_2.JPEG]

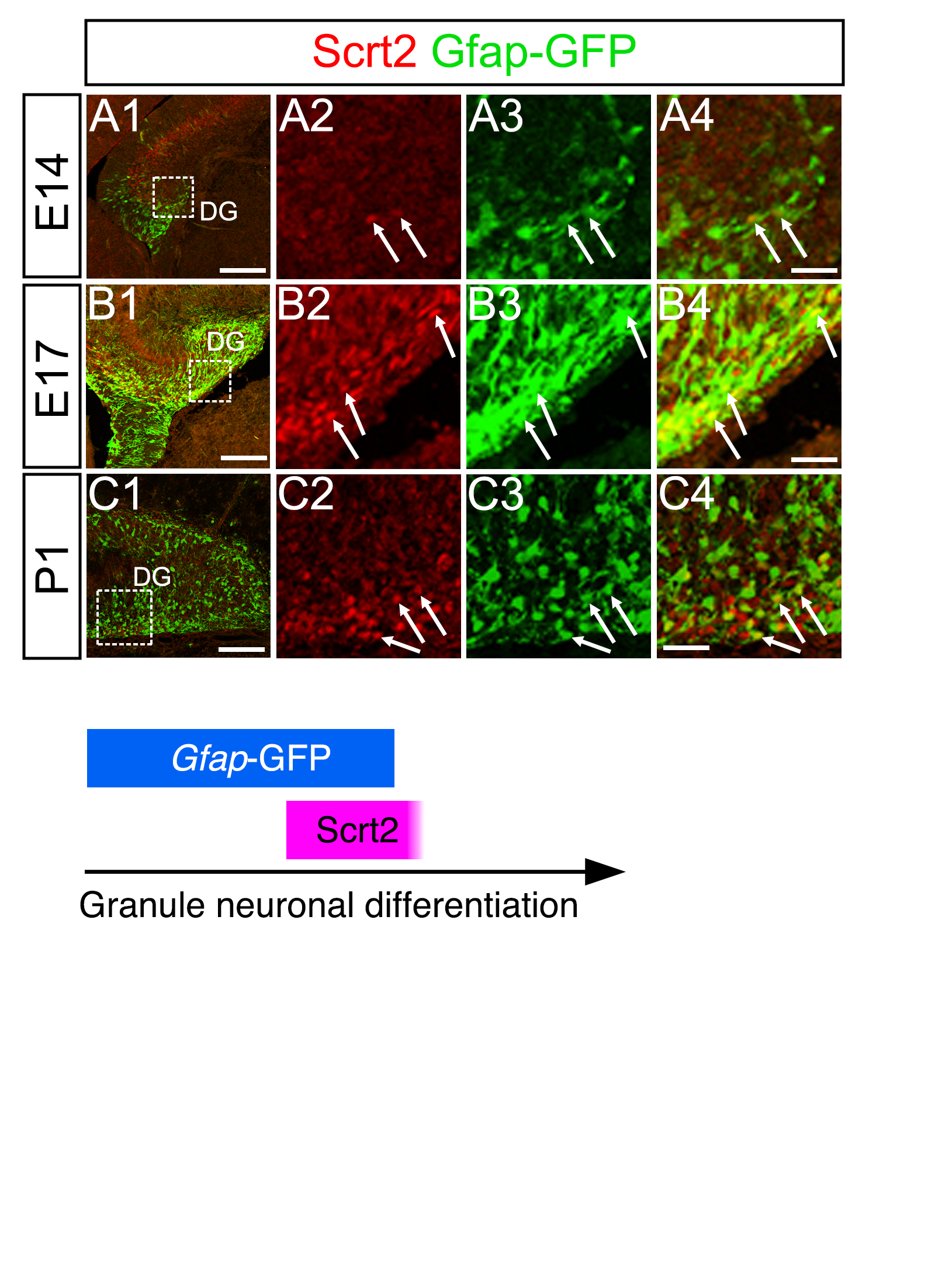

Supplement: SUPPLEMENTARY Figure S3 — Scrt2 expression in gfap-GFP+ DG progenitors. Scrt2 is expressed in gfap-GFP+ DG progenitors at E14, E17, and P1 (arrows in A2–A4,B2–B4,C1–C4, respectively). The box in panels (A1,B1,C1) indicates the region shown in panels (A2–A4,B2–B4,C2–C4). Schematic drawing shows the expression patterns of gfap-GFP and Scrt2 during the differentiation of GNPs. Scale bars; 200 mm in (A1,B1,C1); 50 mm in (A2–A4,B2–B4,C2–C4). [file Image_3.JPEG]

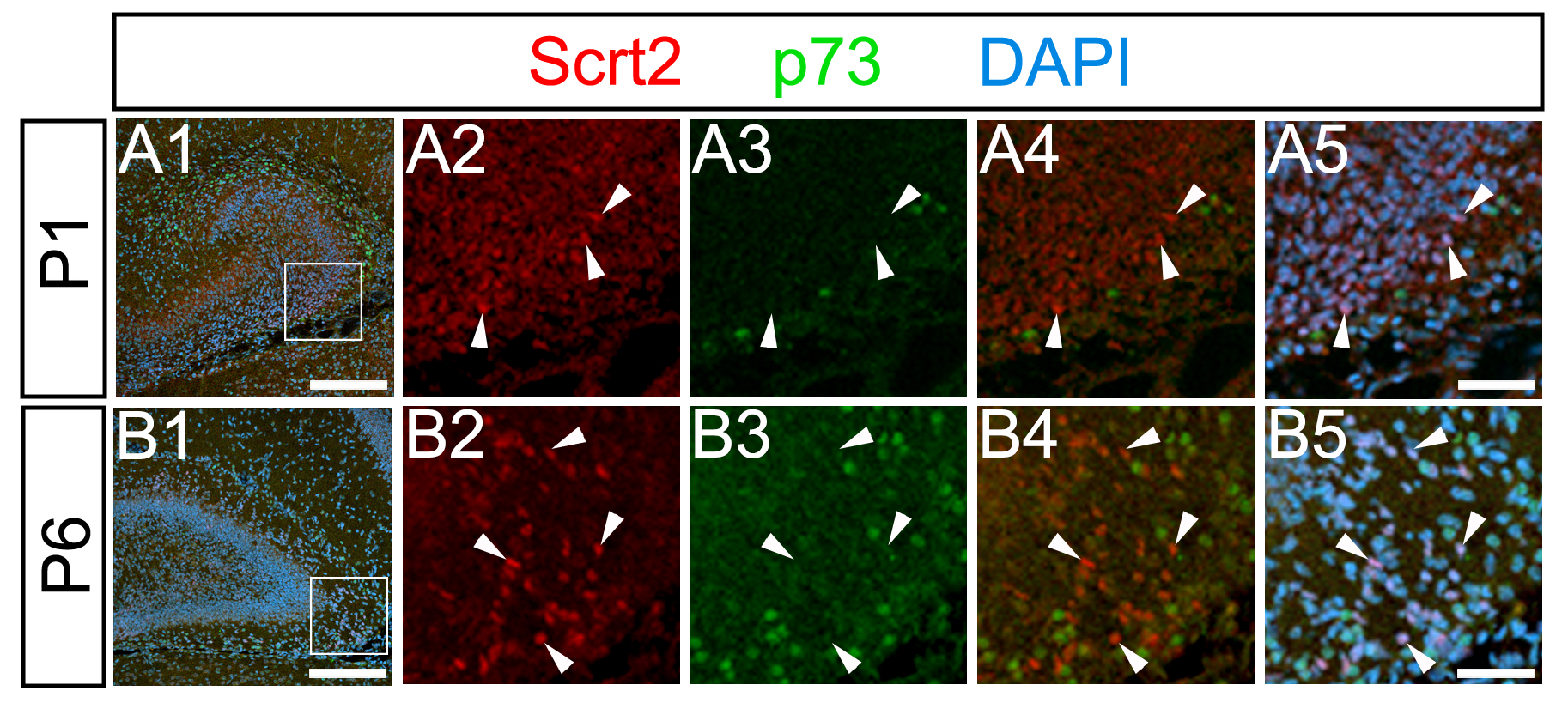

Supplement: SUPPLEMENTARY Figure S4 — Scrt2 is not expressed in p73+ Cajal-Retzius neurons. Scrt2 is not expressed in p73+ Cajal-Retzius neurons at P1 and P6 (arrowheads in A2–A5,B2–B5, respectively). The box in panels (A1,B1) indicates the region shown in panels (A2–A5,B2–B5). Scale bars; 200 mm in (A1,B1); 50 mm in (A2–A5,B2–B5). [file Image_4.JPEG]
